# A strategic assessment of cervical cancer prevention and treatment services in 3 districts of Uttar Pradesh, India

**DOI:** 10.1186/1742-4755-2-11

**Published:** 2005-12-08

**Authors:** Rasha Dabash, Jyoti Vajpayee, Martha Jacob, Ilana Dzuba, Nisha Lal, Jan Bradley, LB Prasad

**Affiliations:** 1 Santa Monica, California, USA; 2 EngenderHealth, New Delhi, India; 3 EngenderHealth, New York, USA; 4 Family Welfare, Uttar Pradesh, India

## Abstract

**Background:**

Despite being a preventable disease, cervical cancer claims the lives of almost half a million women worldwide each year. India bears one-fifth of the global burden of the disease, with approximately 130,000 new cases a year. In an effort to assess the need and potential for improving the quality of cervical cancer prevention and treatment services in Uttar Pradesh, a strategic assessment was conducted in three of the state's districts: Agra, Lucknow, and Saharanpur.

**Methods:**

Using an adaptation of stage one of the World Health Organization's *Strategic Approach to Improving Reproductive Health Policies and Programmes*, an assessment of the quality of cervical cancer services was carried out by a multidisciplinary team of stakeholders. The assessment included a review of the available literature, observations of services, collection of hospital statistics and the conduct of qualitative research (in-depth interviews and focus group discussions) to assess the perspectives of women, providers, policy makers and community members.

**Results:**

There were gaps in provider knowledge and practices, potentially attributable to limited provider training and professional development opportunities. In the absence of a state policy on cervical cancer, screening of asymptomatic women was practically absent, except in the military sector. Cytology-based cancer screening tests (i.e. pap smears) were often used to help diagnose women with symptoms of reproductive tract infections but not routinely screen asymptomatic women. Access to appropriate treatment of precancerous lesions was limited and often inappropriately managed by hysterectomy in many urban centers. Cancer treatment facilities were well equipped but mostly inaccessible for women in need. Finally, policy makers, community members and clients were mostly unaware about cervical cancer and its preventable nature, although with information, expressed a strong interest in having services available to women in their communities.

**Conclusion:**

To address gaps in services and unmet needs, state policies and integrated interventions have the potential to improve the quality of services for prevention of cervical cancer in Uttar Pradesh.

## Background

Cancer of the cervix is preventable, yet approximately 493,100 new cases and more than 273,000 deaths occur each year among women worldwide. [1] While evidence of effective screening programs can be seen throughout the developed world, the burden and impact of the disease remains high in developing countries, where 85% of disease related deaths occur [1].

India, which accounts for one sixth of the world's population, also bears one fifth of the world's burden of cervical cancer [2]. There are approximately 130,000 new cases of cervical cancer in India per year and the disease is reported to be responsible for almost 20 percent of all female deaths there [1]. India's cervical cancer age-standardized incidence rate (30.7 per 100,000) and age-standardized mortality rate (17.4 per 100,000) are the highest in South Central Asia [1]. Data from Mumbai suggest that there may have been a slight decline in cervical cancer incidence in recent years. However, the absolute incidence is still very high, especially in rural areas, and the number of cases grows due to high population growth [2]. Simultaneously, there is also evidence that India is on the verge of a large HIV epidemic [3]. The Indian National AIDS Control Organization estimates that the number of people living with HIV is approximately 5.1 million (38% of whom are women)[4] This suggests cause for concern given the strong association between HIV and HPV infections and evidence of more rapid progression of HPV infections to cervical neoplasia in HIV infected women [5].

Global evidence demonstrates that the key to reducing cervical cancer morbidity and mortality is early detection coupled with timely treatment of cervical precancerous lesions [6]. Cervical cytology often referred to as the *Pap smear *is perhaps the most well known of available screening methods. However, newer screening techniques such as visual inspection methods and HPV-DNA testing have also demonstrated potential for early detection in many settings. These technologies are currently being assessed by the Alliance for Cervical Cancer Prevention (ACCP) for their use in developing countries. As critical as detection is, the need for women with positive results to receive adequate and timely treatment for dysplasia, is paramount. Even in low resource settings, treatment can be offered using low morbidity outpatient procedures such as cryotherapy or Loop Electrosurgical Excision Procedure (LEEP)[7].

With early detection and timely treatment in mind, the World Health Organization recommends that resource poor nations screen all women at least once in a lifetime with priority given to women at the age of 35–40 when likely-to-progress, high- grade but treatable dysplasia can be found [8]. There is some debate about whether this age limit is too high, [9] especially in countries with high HIV incidence, although recent data from South Africa, including 20,000 women showed the mean age of cancer to be 51.6 years [10]. In India in 2003, 46.6% of HIV cases in women occurred in the 15–29 years age group [4]. As in many developing countries and resource-limited settings, India does not have a national vertical or integrated program for screening asymptomatic women and treatment of precancerous lesions. Some Indian states offer ad hoc cervical cancer prevention and treatment services; however, the quality, including access to such services, varies largely by region partly due to the absence of related policies and standards for providing those services.

Uttar Pradesh is India's most populous state. The General Directorate of Family Welfare in the Uttar Pradesh under the Ministry of Health and Family Welfare in collaboration with EngenderHealth, a non-governmental reproductive health organization and member of the Alliance for Cervical Cancer Prevention conducted a strategic assessment on the quality of cervical cancer prevention and treatment services in three of the state's districts: Agra, Lucknow, and Saharanpur. This article summarizes the process and findings of the assessment and presents strategies and recommendations proposed by the stakeholders.

## Methods

The assessment was designed to evaluate the quality and accessibility of cervical cancer prevention and treatment services and was conducted from March to August 2004. The assessment used an adaptation of the first stage of the Strategic Approach to Improving Reproductive Health Policies and Programmes [11], an approach first used to assess the quality of cervical cancer services in Bolivia in 2002 [12]. Guiding this strategic assessment process were these three strategic questions:

1. Is it necessary to introduce policies and interventions for screening, diagnosis and treatment of precancerous cervical lesions in Uttar Pradesh?

2. Is it necessary to improve the quality of information management systems and cancer registries for the prevention, diagnosis, and treatment of cervical cancer in Uttar Pradesh?

3. Is it necessary to improve services for the treatment of cervical cancer in Uttar Pradesh?

The available literature was reviewed and field data were collected during three consecutive weeks by a 30-person multi-disciplinary team of stakeholders. The team included representatives from the Uttar Pradesh Directorate General of Family Welfare, policy-makers, administrators, statisticians, social scientists, researchers, gynecologists, pathologists, public health specialists, cytotechnicians, NGO representatives and community advocates. Data on services were drawn from observations of 94 sites visited, including public, military and private sectors facilities (Table 1). In-depth interviews and focus group discussions were conducted with individuals from key respondent groups, including clients, community members, providers, and policy makers (Table 2). A 10-member Technical Advisory Group guided the technical aspects of this assessment. Observations were conducted at sites where cervical cancer services were offered or could potentially be offered as part of reproductive health services. Using a combination of purposive and snowball sampling participants and health facilities were identified across 18 communities, including urban, peri-urban, and rural communities. Based on the analysis of data from the three districts, responses to the strategic questions were formulated and presented to a wider audience at a meeting in Lucknow. Findings were framed according to their relationship with recognized elements of quality of cervical cancer prevention and treatment programs and services [13,14]; 1) availability, organization and capacity of services; 2) access to services; 3) community knowledge; 4) knowledge and practices of health service providers; 5) training and professional development of providers; 6) quality improvement, including supervision and monitoring; and 7) information systems and cancer registries.

**Table 1:** Number of Sites Visited in all 3 Districts by Sector.

**Public Sector**	
• Specialized Cancer Centers	3
• Tertiary Hospitals	2
• Second Referral Sites (District Hospital)	7
• First Referral Sites (Community and primary health centers)	15
• Maternal and Child Health Units	3
• Laboratories	5

**Private Sector**	

• Private Doctors, Clinics, Nursing Homes, Hospitals	21
• Private Laboratories	19
• Community, Faith-based, and Non-Governmental Organizations	10
• Other	6

**Military Sector**	

Military Hospitals	3

**TOTAL SITES**	**94**

**Table 2:** Sampling by Respondent Category (N = 1197 respondents)

**REPRODUCTIVE HEALTH SERVICES CLIENTS**	
*Female *RH Clients (FGD and IDI)	176
*Male RH Clients (FGD and IDI)*	35
Women with Cervical Cancer (IDI)	9

**COMMUNITY MEMBERS**	

Women (FGD)	319
Men (FGD)	58
Community leaders/Influentials (IDI and FGD)	74

**POLICY INFLUENTIALS**	

Policymakers, health authorities, health program directors (IDI)	71

**PROVIDERS (Interviews and Focus Group Discussions)**	

Obstetricians/Gynecologists (IDI)	45
Other Physicians, Female Medical Officers, Medical Students, Interns (IDI and FGD)	136
Radiotherapists, technicians (IDI)	10
Nurses, Nursing Students, Paramedical Staff (IDI and FGD)	129
Chemists, Registered Medical Practitioners, Traditional Healers (IDI and FGD)	48
Researchers, Statisticians, Demographers (IDI)	14
Cytopathologists and Laboratory Personnel (IDI)	73

IDI – In-depth interviews

FGD – Focus group discussions

## Results

No state-based epidemiological data or policy on cervical cancer prevention exists in Uttar Pradesh. Population-based incidence and mortality data from surveillance sites outside of Uttar Pradesh suggest that cervical cancer is the primary or secondary cause of female related cancer deaths in several urban centers, and may even be higher in rural districts [15].

The strategic assessment identified some isolated cervical cancer prevention and treatment services; however, well-coordinated prevention and stage-appropriate treatment services, particularly in the public sector and outside large urban centers, were lacking. The quality, cost and accessibility of the services varied according to the providing sector (public, private or military) and facility.

### Availability, organization, and capacity of services

#### Screening for cervical precancer and cancer

There was no routine screening offered to asymptomatic women in health care facilities, with the exception of a few private providers and military hospitals. Those providers reported routinely screening asymptomatic women above 35 years in three years interval by using Pap smears. In the public sector, Pap smears were mostly limited to the tertiary care level on an outpatient basis, and usually only offered to women with symptoms of reproductive tract infections or advanced cervical cancer. In the public and private sector, women usually first received a pelvic examination by their gynecologists and were then either referred to the hospital's pathology department or to a private pathology laboratory for their Pap smear.

#### Cytopathology services

Laboratories showed large variation in the procedures used for sample collection, processing, reading and reporting of Pap smears. Few of the laboratories visited were accredited for cytopathology, as required by the Indian Association of Cytopathology (IAC). Mostly private laboratories were unfamiliar with the IACs norms for cytopathology services. Most sites were processing a small number of Pap smears but had the capacity to handle a significantly larger load if provided with additional resources, principally, trained staff and supplies.

#### Diagnosis and treatment of precancer

Equipment and services for diagnosis and treatment of precancerous lesions, including colposcopes and cryotherapy were readily available in military sector facilities and a few private facilities, yet rarely available or functioning at public tertiary sites. Some sites had cryotherapy equipment but were only using it to treat 'cervical erosion'; few staff at these sites knew that it could be used to treat cervical precancer. Thus, in the public sector and in many private facilities, invasive inpatient procedures such as hysterectomy were the first line of treatment and management of women with precancer, even for mild dysplasia.

#### Cervical cancer treatment, including palliative care

Most women with cervical cancer were diagnosed at later stages of the disease. Uttar Pradesh has 11 well-equipped cancer treatment facilities. Radical surgery for treatment of early stages of cancer and radiotherapy for later stages, were available for private and military sector patients, but was usually financially and geographically inaccessible for public sector patients in two of the three districts. Frequently, women using public services had to travel to other districts in Uttar Pradesh or to New Delhi to receive cancer treatment. Palliative care, including opioid treatment for pain relief, for terminally ill women was even less accessible. Only one non-governmental organization had recently started providing palliative care services in one of the assessment districts.

#### Factors influencing access to services

In addition to the limited numbers of highly centralized services (Table 3), there were several factors affecting women's access to cervical cancer prevention and treatment services. Where offered, the poor organization of services in the public sector (often depending on a single provider's presence), translated into women having to make numerous visits and increasing therefore the likelihood of loss to follow-up.

**Table 3:** Availability of Cervical Cancer Services in the Public Sector in Uttar Pradesh

***Health Care Level***	***Description***	***Services Available***
**First Referral Sites**	Rural community health centers, Primary health centers, and rural sub-centers	Only referrals of women with reproductive tract infections symptoms to district hospitals for Pap smears.
**Second Referral Sites**	Urban district hospitals	A few Pap smears performed on symptomatic women, treatment of precancer (cryotherapy* available but hysterectomy was most often used). In many secondary referral sites, women just referred to tertiary facilities.
**Tertiary Hospitals**	Urban Medical Colleges and Universities	Pap-tests, colposcopy, Treatment of precancer (cryotherapy* available but hysterectomy was most commonly performed), cancer treatments (radical surgery, radiotherapy or chemotherapy).
**Specialized Oncology Treatment Centers (Under National Cancer Control Program)**	11 in Uttar Pradesh state, with one in Lucknow; none in Agra or Saharanpur	A range of cancer treatment services, including surgery, radiotherapy, chemotherapy.

*Cryotherapy equipment and supplies were available in some sites; however, they were mostly used for treating "cervical erosion" but not for the treatment of precancerous lesions.

The costs of services varied considerably by site and sector (Table 4), but even in the public sector, consultation fees coupled with the often high indirect costs of having to seek services (e.g. transportation, lost wages, long waits) were reported to negatively influence access. As one client interviewed said, *"the hospital is very far from my home. So it takes a very long time and costs about Rs. 80 in transportation." *For many, this equivalent of two days wages was often too costly.

**Table 4:** Average Cost in Rupees ($1 = 45 Rupees) by Sector of Various Cervical Cancer Prevention and Treatment Services

***Type of Service***	***Public***	***Private (For-profit and NGOs)***	***Military***
**Consultation**	1	1–300	Free or minimal charge
**Pap Collection and Cytology**	0–13	36–300	Free or minimal charge
**Colposcopy**	50	500	Free or minimal charge
**Cryotherapy/LEEP**	50	500	Free or minimal charge
**Cervical Biopsy**	50	150–250	Free or minimal charge
**Hysterectomy**	400	10,000–25,000	Free or minimal charge
**Radiotherapy**	900–5000	7,200–20,000	Free or minimal charge
**Radical Surgery**	400	25,000–40,000	Free or minimal charge

Equally important, negative perceptions of the community and clients about the quality of public sector services were also reported to discourage clients from attending, especially in the absence of symptoms. On client asked *"if I do not have any pain or any other symptoms, why should I go for examination?" *These factors were often compounded by the socio-cultural notions challenging women's comfort with having a pelvic examination, especially by a male provider.

Issues of confidentiality and privacy were not just barriers to women getting screening but were a reality for women diagnosed with cervical cancer. As one woman undergoing radiotherapy said, "*The hardest part of this is that I have lost my dignity. What was once private, I now have to discuss with the doctor in front of my son*." Male relatives were often reported to play a key role as facilitators or gatekeepers to women's access to services. Finally, given the limited financial means of many community members, the value of women's health and prevention of illness were often reported as secondary to competing financial and social responsibilities. As one provider explained, "*for many of the patients, meeting daily needs is more crucial; preventive care and detecting cervical cancer is not the priority*."

#### Community knowledge about cervical cancer

Information, Education and Communication (IEC) campaigns on breast, prostate and oral cancers had raised awareness in communities about the causes and prevention of these diseases. This was not the case for cervical cancer for which virtually no IEC materials or efforts promoted its preventable nature in the vast majority of communities and facilities visited. Where available, counseling in tertiary facilities for women was often inadequate or incomplete. Most community members reported familiarity with other forms of cancer, but rarely knew of cervical cancer. Only very few community members and clients who knew someone who had been diagnosed with cervical cancer reported some familiarity with the disease, but also did not know that it was preventable.

#### Women's knowledge and perceptions

Few community members or asymptomatic women had ever been screened for cervical cancer. Those who ever had a Pap smear or were interviewed when they presented for screening also did not know that the test could help in preventing cervical cancer. Similarly, women interviewed who were undergoing treatment for early and late stage cervical cancer were often unaware of their exact diagnosis or if aware, did not know the cause of the disease for which they were being treated.

*"I only know cancer by name. It is a disease which cannot be treated. So a person dies. My grandmother had some genital cancer about 5–6 years back. She was treated, but ultimately she died*. For many, including this female community member, cervical cancer was commonly equated with death. *"I am ashamed of this disease in this old age," *said one woman interviewed who was undergoing radiotherapy for cervical cancer. Since many believed that cervical cancer was caused by poor hygiene, high parity, promiscuity, fibroids, and/or use of contraceptive pills or IUCD, many reported fearing that a cervical cancer diagnosis would bring a woman shame, blame and even abandonment by her husband and family. Given such stigma, most women with cervical cancer had not been told about the nature of their disease, mostly at the request of family members. Some women believed that cervical cancer is caused by pelvic examination; this belief was based on the perception that it could activate or exacerbate dormant cancer cells.

#### Men's knowledge

The perceptions of men from the community and male clients were similar to those of women. Like women, male respondents interviewed also had large knowledge gaps and misconceptions about the cause of cervical cancer. Some men reported discomfort discussing matters of reproductive health or sexuality with women; yet, after having learned the potential benefits of screening, most were willing to encourage their wives and female relatives to be screened. Most male clients at the military hospitals were aware that women above the age of 35 years should have regular examinations and undergo tests, but they were not sure about why the tests were being performed and did not know that cervical cancer is preventable.

#### Awareness among community leaders

Likewise, community leaders, while generally unaware of the cause and magnitude of cervical cancer, were particularly interested in learning how they could facilitate prevention and awareness, particularly IEC and screening campaigns. Many stressed the importance of access to these services, particularly at the primary health care level. One community leader said, *"I will make cancer cervix on the priority list. I will be the part of the team to make the community aware about the disease. I will involve ANMs (midwives) to visit house to house so that they can talk to women one on one basis. But the program should come to our community."*

### Providers' knowledge and practices

Many providers knew about the link between cervical cancer and Human Papilloma Virus (HPV), yet gaps in understanding the natural history of cervical cancer, its preventable nature, treatment of precancerous lesions, and knowledge of stage-appropriate clinical management of cancer were present. Knowledge about cervical cancer was generally better at higher-level facilities and in the private and military sectors. Generally, more physicians were aware about cervical cancer issues than mid-level and paramedical staff. It was not uncommon that doctors were unaware of the long period during which precancerous lesions could be detected and treated; which may explain why few doctors were performing routine screening of asymptomatic women or targeting younger women. Military sector providers were generally more knowledgeable about the importance of age-targeted interval screening and population coverage strategies to reduce cervical cancer morbidity and mortality.

While familiar with Pap smears, few providers had knowledge of alternative screening approaches, such as HPV-testing and visual inspection methods (e.g. visual inspection with acetic acid (VIA) or visual inspection with Lugol's iodine (VILI)). Most gynecologists in public and private facilities felt competent in taking cervical samples for Pap smears but said that they generally referred clients to pathology labs for the pathologists, residents or female technicians to take the cervical sample. Some reported doing so for their own convenience while others believed that laboratory personnel were better suited to take smears. Observations and provider interviews suggested that at least some of the cervical samples collected were of poor quality due to noted gaps in provider practices, such as using cotton swabs to collect samples or improperly collecting the cervical smear.

"I treat dysplasia I and II with an antibiotic and antioxidant course for three months then repeat the Pap smear. If it progresses or remains the same, I advise hysterectomy."

Similar to this public sector provider, most doctors did not differentiate between an indication for treating precancerous lesions and cancer, usually performing a hysterectomy to manage most forms of dysplasia. Mid-level and nursing staff rarely knew the difference between cervical precancer and cancer or about stage-appropriate treatments. Additionally, few public sector providers had been trained or had access to colposcopes for cervical cancer diagnosis. Access to and knowledge of the value of simple and effective precancer treatments such as cryotherapy and LEEP were also limited among providers in the public sector, but considerably better among private and military sector providers and public sector oncologists and radiotherapists. Knowledge about palliative care, including pain relief, as a way of providing clinical and psychosocial support for terminally ill patients, was generally poor among most providers.

### Training and professional development of health care providers

Discussion with providers and those responsible for training medical and nursing students revealed that there was limited access to relevant pre-service training programs and few professional development opportunities. Few medical teaching institutions were routinely training gynecology residents to perform Pap smears, colposcopy or cryotherapy. Those responsible for offering cancer treatment generally had formal postgraduate training in radiotherapy. Pathologists generally reported having received postgraduate training in reading cervical smears but had learned to take Pap smears on-the-job. Cervical cancer issues were not included in the theoretical or practical training of nurses. Staff at almost all laboratories reported a shortage of formally trained cytotechnicians. Most laboratories were instead relying on in-house trained technicians.

There were few opportunities for continuing professional development in reproductive female oncology. The Federation of Obstetric and Gynecological Society of India had recently published a review of screening methods and approaches, but few gynecologists were even aware of this resource. Similarly, national organizations such as the Indian Medical Association and the Indian Association of Cytopathology were rarely mentioned as sources of information on cervical cancer issues.

### Quality improvement and quality assurance

Functioning quality improvement systems, which include effective supervision and monitoring, are key aspects of any health program. Internal and external laboratory quality assurance systems were not available in most laboratories. Most ad-hoc cervical cancer services were not based on any official policy and were generally integrated into the services provided by several departments (gynecology, pathology, and oncology) without clear guidance and indicators of what constituted quality and how it should be monitored. Although some facilities initially reported that they had internal quality assessment systems, there was little evidence of these in most sites visited.

### Information systems and cancer registries

Firstly, cancer registries contain data that can be used to track and follow-up patients. Secondly, they can quantify the magnitude of morbidity and mortality associated with the disease. In Uttar Pradesh, neither information systems nor cancer registries existed. Even facility-based data, collected routinely by districts on infectious and communicable diseases did not include any indicators or data on cervical cancer.

A few private and military sites were found to be using high tech computerized systems to capture client data for tracking and billing, but more commonly non-standardized manual registries were used by the sites. Referrals and follow-up were generally presumed to be the patient's responsibility, and few sites tracked those with positive Pap smear results. At some sites as many as 80% of women were said to have failed to return for their results. Follow-up was rarely conducted to track clients with cervical precancer or cancer.

## Discussion

These findings reveal a large need for the expansion of cervical cancer prevention and treatment services in the public and private sectors. To develop cost-effective strategies for improving quality and increasing access, responses to the three strategic questions were considered by the stakeholders:

### Is it necessary to introduce policy and interventions for screening, diagnosis, and treatment of precancerous cervical lesions?

While there were some services providing screening and treatment of cervical precancerous lesions, staff was often managing services with little guidance. National guidelines on cervical cancer prevention and management should be provided. Policies should be considered in the light of multiple competing health priorities in Uttar Pradesh and should build on existing resources and services, provider and system capacities, and community perceptions. State-level policy should include an emphasis on pre-service medical education. Professional institutions may be the key to reach existing and future generations of providers with the knowledge and skills needed to deliver effective services. This assessment confirmed findings from studies in similar settings that many providers were unaware of the natural history of HPV infection and the appropriate timing and frequency of screening interventions. Orientation of international guidelines on target age groups, screening frequency and the need for increased coverage is urgently needed.

To address the lack of awareness and knowledge among providers, clients and the community at large, IEC materials about cervical cancer and its prevention should be developed and disseminated along with information for women over 35 years old on where and how to get screened. Simultaneously, interventions are needed to meet the demand by maximizing the capacity of existing technologies and resources with linkages to the primary community level. In particular, program planners should look at the possibility of providing counseling and screening for cervical cancer in the voluntary counseling and testing centers for HIV (VCT), of which there are currently 70 in Uttar Pradesh [4].

### Is it necessary to improve the current information system and cancer registry for the prevention, diagnosis and treatment of cervical cancer?

Since screening without the possibility for treatment would be pointless, functional mechanisms for tracking women for follow-up care need to be introduced. As suggested by community leaders, NGOs and community groups should be engaged to play a key role in these aspects, provided attention is paid to confidentiality and clients' rights. Opportunities for collecting population-based data on coverage, morbidity and mortality should also be considered as interventions to be integrated into reproductive health services. The potential of linking with state-based and national information systems to collect these data should also be explored.

### Is it necessary to improve the existing services for the treatment and management of cervical cancer?

Given that there are 11 well-equipped cancer treatment centers in Uttar Pradesh, stronger linkages and referral systems to these facilities are needed, particularly for women in districts with limited services. The costs of transport and lodging for women and their families could potentially be offset by community and NGO supported mechanisms. Within these facilities and at the community level, emphasis should also be given to palliative care for terminally ill women.

## Conclusion

As prophylactic and therapeutic HPV vaccines are still under development, and evidence that other primary prevention measures are not useful [16], secondary prevention, in the form of screening and treatment of precancerous lesions continues to be the most effective way to reduce the incidence of cervical cancer at the present time. Findings of the strategic assessment reveal the need for policies and interventions to expand women's access to cervical screening and treatment facilities. In his regard, India is not different from other low resource settings, where programs are lacking or failing due to inadequate legislative and regulatory frameworks, poor quality services, outdated knowledge and uninformed consumers. As in other settings, political will and resources, need to be made available. Even without large resources, it is possible to develop programs by assessing needs, re-assigning existing resources more wisely and creating synergies between different stakeholders. For example, it is useful to focus limited staff and laboratory resources on screening women in key age groups, rather than on very young women. It might also be useful to create new linkages with services that focus on older women, for example, hypertensive clinics, or those who manage clients at high risk of contracting HIV.

## Competing interests

The author(s) declare that they have no competing interests.

## Authors' contributions

RD coordinated the research process, participated in all aspects of data collection and analysis and authored this manuscript.

JV coordinated the assessment in India with the Directorate General of Family Welfare, participated in the assessment process and provided feedback on this manuscript.

MJ provided clinical and research leadership during the assessment, participated in all aspects of the assessment and provided input on this manuscript.

ID provided programmatic and technical input to the assessment and input on this manuscript.

NLparticipated in the strategic assessment and provided input on this manuscript.

JBprovided technical support to the planning of the assessment and technical input on the final report and this manuscript.

LBP facilitated the assessment coordinated process in Uttar Pradesh, served as a member of the technical advisory group and reviewed assessment findings and this manuscript.
